# Early-childhood body mass index and its association with the COVID-19 pandemic, containment measures and islet autoimmunity in children with increased risk for type 1 diabetes

**DOI:** 10.1007/s00125-023-06079-z

**Published:** 2024-01-12

**Authors:** Sandra Hummel, Sarah Rosenberger, Thekla von dem Berge, Rachel E. J. Besser, Kristina Casteels, Angela Hommel, Olga Kordonouri, Helena Elding Larsson, Markus Lundgren, Benjamin A. Marcus, Mariusz Oltarzewski, Anne Rochtus, Agnieszka Szypowska, John A. Todd, Andreas Weiss, Christiane Winkler, Ezio Bonifacio, Anette-G. Ziegler, for the GPPAD and POInT Study Group

**Affiliations:** 1 Institute of Diabetes Research, Helmholtz Munich, German Research Center for Environmental Health, Munich, Germany; 2 Forschergruppe Diabetes e.V. at Helmholtz Zentrum München, Munich, Germany; 3 School of Medicine, Forschergruppe Diabetes at Klinikum rechts der Isar, Technical University Munich, Munich, Germany; 4 Institute for Medical Information Processing, Biometry and Epidemiology - IBE, Ludwig-Maximilians-Universität München, Munich, Germany; 5 Pettenkofer School of Public Health, Munich, Germany; 6 Kinder- und Jugendkrankenhaus auf der Bult, Hannover, Germany; 7 Centre for Human Genetics, JDRF/Wellcome Diabetes and Inflammation Laboratory, Nuffield Department of Medicine, NIHR Biomedical Research Centre, University of Oxford, Oxford, UK; 8 Department of Pediatrics, University Hospitals Leuven, Leuven, Belgium; 9 Department of Development and Regeneration, KU Leuven, Leuven, Belgium; 10 Center for Regenerative Therapies Dresden, Technische Universität Dresden, Dresden, Germany; 11 Paul Langerhans Institute Dresden of the Helmholtz Munich at University Hospital Carl Gustav Carus and Faculty of Medicine, Technische Universität Dresden, Dresden, Germany; 12 Unit for Pediatric Endocrinology, Department of Clinical Sciences Malmö, Lund University, Lund, Sweden; 13 Department of Paediatrics, Skane University Hospital, Malmö/Lund, Sweden; 14 Department of Pediatrics, Kristianstad Hospital, Kristianstad, Sweden; 15 Department of Paediatric Diabetology and Paediatrics, The Children’s Clinical Hospital Józef Polikarp Brudziński, Warsaw, Poland; 16 Department of Paediatrics, Medical University of Warsaw, Warsaw, Poland

**Keywords:** Childhood BMI, COVID-19, Islet autoimmunity, Stringency index, Type 1 diabetes

## Abstract

**Aims/hypothesis:**

The aim of this study was to determine whether BMI in early childhood was affected by the COVID-19 pandemic and containment measures, and whether it was associated with the risk for islet autoimmunity.

**Methods:**

Between February 2018 and May 2023, data on BMI and islet autoimmunity were collected from 1050 children enrolled in the Primary Oral Insulin Trial, aged from 4.0 months to 5.5 years of age. The start of the COVID-19 pandemic was defined as 18 March 2020, and a stringency index was used to assess the stringency of containment measures. Islet autoimmunity was defined as either the development of persistent confirmed multiple islet autoantibodies, or the development of one or more islet autoantibodies and type 1 diabetes. Multivariate linear mixed-effect, linear and logistic regression methods were applied to assess the effect of the COVID-19 pandemic and the stringency index on early-childhood BMI measurements (BMI as a time-varying variable, BMI at 9 months of age and overweight risk at 9 months of age), and Cox proportional hazard models were used to assess the effect of BMI measurements on islet autoimmunity risk.

**Results:**

The COVID-19 pandemic was associated with increased time-varying BMI (*β* = 0.39; 95% CI 0.30, 0.47) and overweight risk at 9 months (*β* = 0.44; 95% CI 0.03, 0.84). During the COVID-19 pandemic, a higher stringency index was positively associated with time-varying BMI (*β* = 0.02; 95% CI 0.00, 0.04 per 10 units increase), BMI at 9 months (*β* = 0.13; 95% CI 0.01, 0.25) and overweight risk at 9 months (*β* = 0.23; 95% CI 0.03, 0.43). A higher age-corrected BMI and overweight risk at 9 months were associated with increased risk for developing islet autoimmunity up to 5.5 years of age (HR 1.16; 95% CI 1.01, 1.32 and HR 1.68, 95% CI 1.00, 2.82, respectively).

**Conclusions/interpretation:**

Early-childhood BMI increased during the COVID-19 pandemic, and was influenced by the level of restrictions during the pandemic. Controlling for the COVID-19 pandemic, elevated BMI during early childhood was associated with increased risk for childhood islet autoimmunity in children with genetic susceptibility to type 1 diabetes.

**Graphical Abstract:**

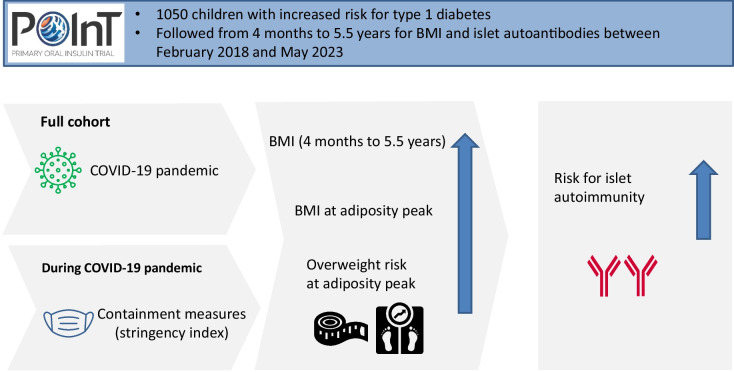

**Supplementary Information:**

The online version of this article (10.1007/s00125-023-06079-z) contains peer-reviewed but unedited supplementary material.


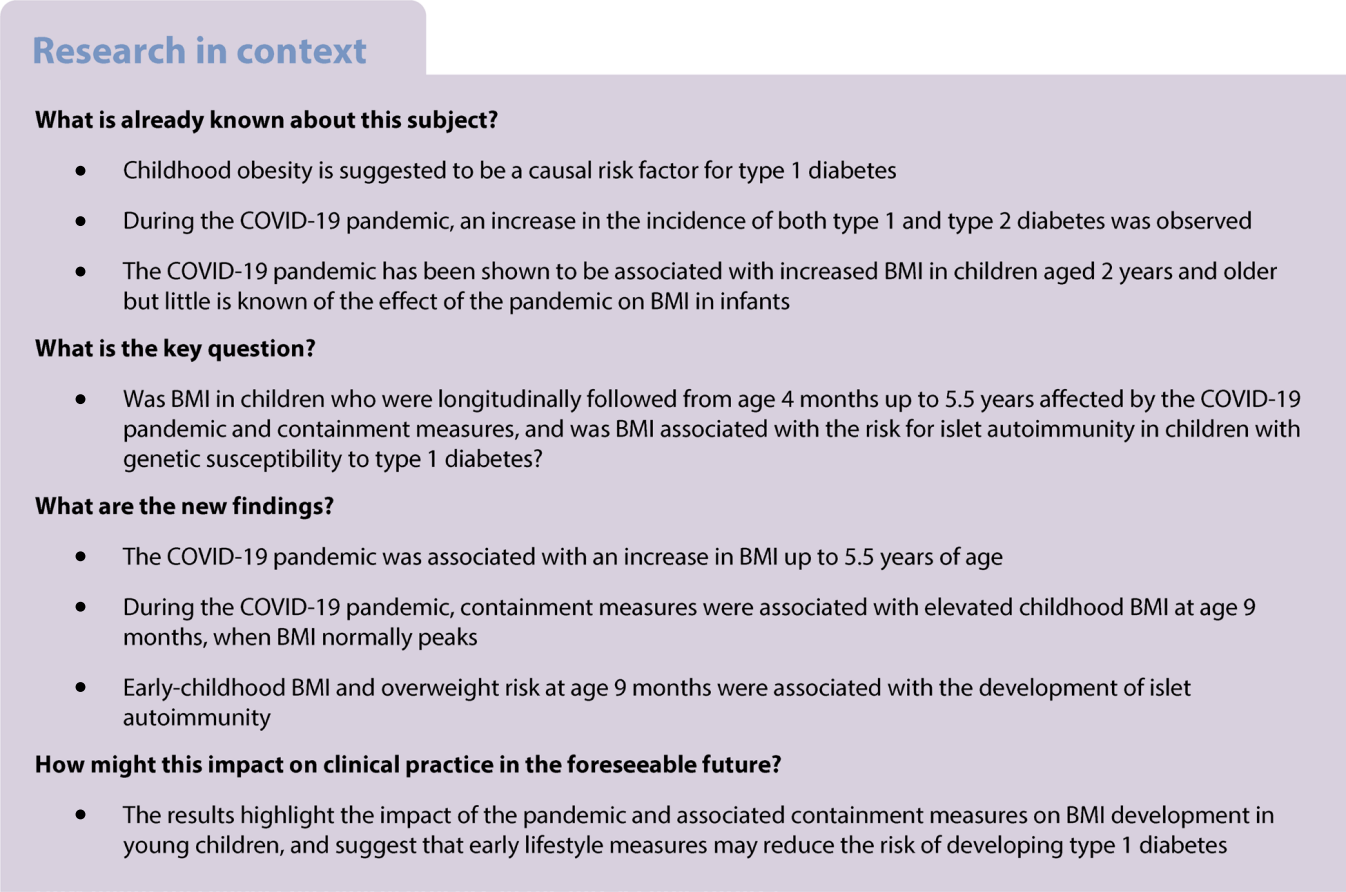


## Introduction

The COVID-19 pandemic has been linked to increases in the incidence of both type 1 and type 2 diabetes [1–3]. SARS-CoV-2 infection may have contributed to the observed increase [1], but lifestyle changes that occurred during the pandemic may also have had an impact on diabetes development. Containment measures implemented by national health authorities in response to the pandemic to prevent the spread of SARS-CoV-2 led to changes in dietary and physical activity behaviours among children and adolescents [4]. As a result, an increased prevalence of weight gain and overweight was observed in children during the COVID-19 pandemic compared with before the pandemic [5, 6]. However, no studies included children below age 2 years, which is the period of highest islet autoantibody seroconversion rate [7, 8]. Indeed, the incidence rate of islet autoantibodies is highest around 1 year of age, which is soon after the BMI peak observed at age 9 months. Moreover, an earlier age at BMI peak and a higher growth rate around the BMI peak have been shown to be associated with several health outcomes, including an increased risk for islet autoimmunity [9, 10]. Therefore, increases in BMI and growth rate in infancy and early childhood may have relevance to the development of islet autoimmunity and future type 1 diabetes. The aim of this study was to determine whether the COVID-19 pandemic was associated with increased BMI and overweight risk around the age of the BMI peak, and whether there was an association between BMI and the risk for islet autoimmunity. This was addressed in over 1000 children with genetic susceptibility to type 1 diabetes who were prospectively followed in the multinational Primary Oral Insulin Trial (POInT) between 2018 and 2023 for BMI and islet autoantibody development [11].

## Methods

### Study design

POInT is an ongoing randomised, controlled and multicentre clinical trial organised through the Global Platform for the Prevention of Autoimmune Diabetes (GPPAD), which is being performed to investigate whether daily intake of oral insulin reduces the incidence of islet autoimmunity and/or type 1 diabetes in children with an increased risk of type 1 diabetes [11]. The infants were randomised to receive either oral insulin powder or oral placebo powder daily until 3 years of age. Recruitment started in February 2018, and seven clinical research centres were included: three in Germany (Dresden, Hanover and Munich), one in Sweden (Malmö), one in Poland (Warsaw), one in Belgium (Leuven) and one in the UK (Oxford). Infants were eligible if they had a genetic risk for developing multiple islet autoantibodies of >10% by the age of 6.0 years. A detailed description of the study protocol has been published previously [11].

### Participants

A total of 1050 infants were enrolled at 4.0–7.0 months of age, and followed at 2, 4 and 8 months after the baseline visit, at 1.5 years of age, and then every 6 months until the maximum age of 7.5 years. The present analyses included measurements up to a maximum age of 5.5 years (see electronic supplementary material [ESM] Table 1).

### Growth

The height and weight of the participants were recorded at the study sites by trained personnel at each visit. Height (cm) was measured as length before 2 years of age, and after that as standing height measured to the closest 0.1 cm using a wall-mounted stadiometer. Body weight (kg) was measured using a calibrated electronic scale. Height/length and weight were used to calculate BMI (kg/m^2^). Additionally, height/length and weight were transformed to weight-for-length *z* score values, and BMI was transformed to standard deviation score (SDS) values using age- and sex-specific WHO reference values [12]. BMI SDS values less than −5 or greater than 5 (*n*=9 of 8839 BMI SDS values) were deemed implausible and excluded [13]. BMI SDS values >1 defined children as at risk for overweight [14], and a weight-for-length *z* score >2 defined children as overweight [12].

### COVID-19 pandemic and containment measures

A cut-off date of 18 March 2020, which was the mean start date of the first lockdown in the participating countries and the time at which the European Commission took the first measures against the COVID-19 outbreak, was used to classify BMI measurements as being taken before or during the COVID-19 pandemic. To quantify the potential impact of COVID-19 policy responses taken by governments of the participating study countries after the COVID-19 outbreak, we used the stringency index from the open-access global pandemic policies database, the ‘Oxford COVID-19 Government Response Tracker’ (OxCGRT), which provides systematic measures of government responses by tracking publicly available policies and interventions taken by governments in response to the COVID-19 pandemic in more than 180 countries [15]. The stringency index for any given day is an additive unweighted index representing the intensity of COVID-19 policies applied by national governments. It is based on nine indicators, comprising school and work closures, cancellation and restriction of public events and gatherings, stay-at-home requirements, public information campaigns, restrictions on internal movements, and public transport and international travel controls. All indicators were reported on ordinal scales, and a score between 0 and 100 was created by rescaling the ordinal value. The scores were then averaged to obtain the composite index.

### Genotyping and BMI genetic risk score

SNP data were generated as previously described [16] using the Infinium Global Screening Array (version 3.0, Illumina) on DNA extracted from dried blood spots from children for whom consent to store and use dried blood spots for additional research was provided. A detailed description of the genotyping is provided in ESM Methods 1. A genetic risk score (GRS) was calculated to estimate the combined effect of selected SNPs on early-childhood BMI. The risk score was calculated based on 33 of 46 SNPs that have previously been shown to be associated with infant or early-childhood BMI [17] (ESM Table 2).

### Definition of islet autoimmunity outcome

Serum samples from each visit were analysed for autoantibodies to insulin, GAD65, IA-2 and ZnT8 (ZnT8RA and ZnT8WA) [16]. A detailed description of islet autoantibody measurement is provided in ESM Methods 2. The islet autoimmunity outcome was defined as either development of persistent confirmed multiple islet autoantibodies, which was defined as autoantibodies to insulin, GAD65, IA-2 or ZnT8 in two consecutive samples and a confirmed second islet autoantibody in one sample, or one or more of the antibodies and type 1 diabetes. Maternally transferred islet autoantibodies were identified and excluded if the child was positive at the first sample, had declining antibody titres on follow-up, and subsequently became islet autoantibody-negative. For children classified as islet autoantibody-positive, the seroconversion timepoint was defined as the first confirmed positive sample.

### Study approval

Ethical approval for the POInT study was obtained from local ethical committees and regulatory authorities of the Technische Universität München, Medical Faculty (326/17 Af), the Medical University of Warsaw (Instytucie Matki I Dziecka w Warszawie) (199/2017), the UK Health Research Authority (18/SC/0019), Onderzoek UZ/KU Leuven (S60711) and the Regionala etikprövningsnämnden i Lund (2017/918). The parents or legal representatives of each participant provided written informed consent, and further agreed to biobank storage of material that was used in this study.

### Statistical analysis

Descriptive statistics for continuous variables are presented as median and IQR and those for categorical variables are presented as counts and percentages. The number of individuals included in each component of the analysis is indicated in the relevant tables and figures.

A linear multivariate mixed-effect model was used to identify whether exposure to the COVID-19 pandemic affected the time-varying, longitudinally measured outcome BMI, adjusting for BMI GRS, sex (self-reported), age and country of residence. A binary covariate was included in this model, coded as ‘0’ if a given BMI measurement was obtained before the COVID-19 pandemic, and as ‘1’ if it was obtained during the COVID-19 pandemic, irrespective of how many BMI measurements for a child were obtained before or during the pandemic. Applying an additional linear multivariate mixed-effect model, we also investigated whether longitudinally measured BMI up to 18 months of age was different between children for whom all BMI measurements up to 18 months of age were obtained before or during the COVID-19 pandemic. A binary variable was included in this model, coded as ‘0’ or ‘1’ if these BMI measurements were obtained before or during the COVID-19 pandemic, respectively. Random effects were included in the model in the form of random intercepts at the participant level. For the linear regression models, the assumption that residuals were normally distributed and the assumption of equality of variance were tested.

To assess whether the exposure to the COVID-19 pandemic was associated with BMI and overweight risk at around the age of the BMI peak, the BMI measurement that was taken closest to age 9 months was used as a surrogate measure for the magnitude of the BMI peak [18] in multivariate linear and logistic regression models, adjusted for BMI GRS, sex and country of residence. Interaction terms were added to the model to explore potential effect modification between the COVID-19 pandemic and BMI GRS, sex or country of residence with respect to the outcome overweight risk at 9 months of age.

To assess the effect of the stringency index (a measure of responses to the COVID-19 pandemic) on early-childhood BMI, the analysis was restricted to the cohort of children with BMI measurements obtained during the COVID-19 pandemic. To align the stringency index with the date of BMI measurements, the country-specific stringency index for the respective visit date was selected from the ‘Oxford COVID-19 Government Response Tracker’ [15], and the models were adjusted for BMI GRS, sex and age (linear mixed model) or for BMI GRS and sex (linear and logistic regression models). The results for the effect of the stringency index on BMI are shown per 10-point increase in the stringency index. Similar analyses were performed using BMI SDS and the weight-for-length *z* score as outcome measures.

Associations between age-corrected early-childhood BMI (BMI as a time-varying variable and BMI SDS>1 at 9 months) and the development of islet autoimmunity were analysed using Cox proportional hazard regression models. The proportionality of hazards was evaluated by the log [−log (survival)] vs log (time) graph method. Duration was calculated from the first measurement to the time at first islet autoantibody positivity or the time to last visit in children who did not develop islet autoimmunity during follow-up. To study whether childhood BMI was associated with early islet autoimmunity outcome (until 2.5 years of age), only children who developed the outcome before 2.5 years of age were defined as cases. All models were adjusted for first-degree family history of type 1 diabetes (yes/no), sex, country of residence and whether the BMI measurement was obtained before or during the COVID-19 pandemic. Interaction terms were added to the model to explore potential effect modification between overweight risk at 9 months of age and sex or country of residence with respect to the islet autoimmunity outcome.

All statistical analyses were performed using R statistical software (Austria), version 4.2.0, and an α-level of 0.05 was considered as statistically significant.

## Results

**Fig. 1:**
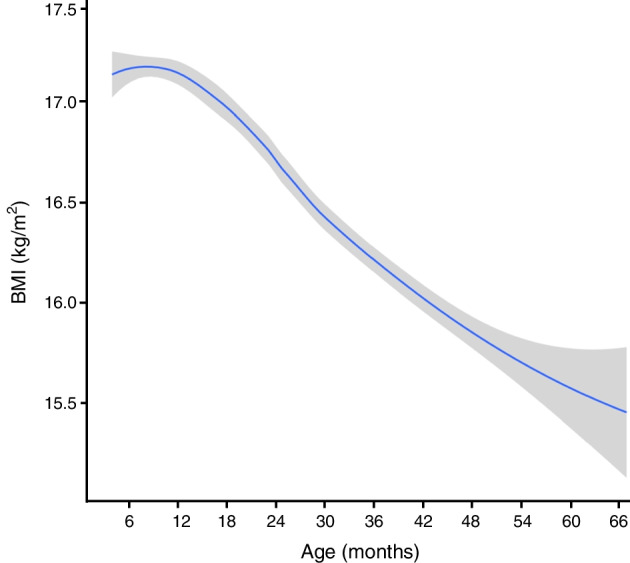
LOESS (locally estimated scatterplot smoothing) regression fitted curve showing variation of BMI in relation to age and 95% CI for 8839 BMI measurements of 1050 children

Growth data were available from 533 boys and 517 girls, including 556 (53.0%) children with a first-degree family history of type 1 diabetes (ESM Table 3). The children had a median age of 0.51 years (IQR 0.45–0.54) at the first measurement (visit 1), and were followed for a median of 2.9 years (IQR 2.4–3.5) to a maximum age of 5.5 years (ESM Table 1). The BMI of the 1050 children by age is shown in Fig. 1 and ESM Fig. 1.

### Effect of the COVID-19 pandemic and containment measures on early-childhood BMI

**Fig. 2:**
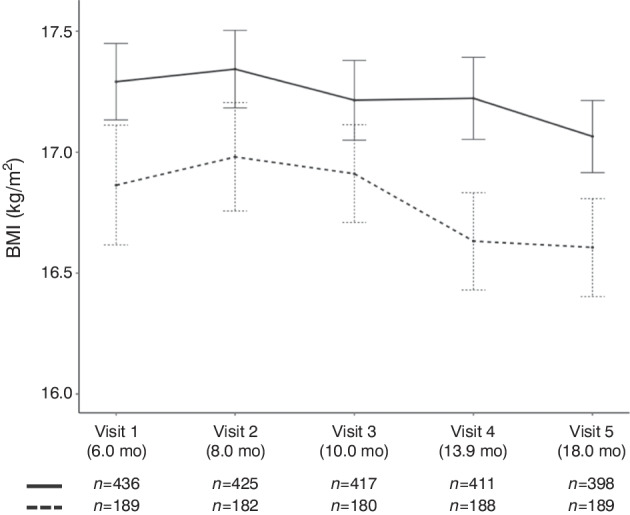
BMI (mean and 95% CI ) for children whose first five visits occurred during the COVID-19 pandemic (solid line) compared with children whose first five visits occurred before the pandemic (dashed line) (linear multivariate mixed-effect model: *β* = 0.51; 95% CI 0.21, 0.81, *p*=0.001; adjusted for first-degree family history of type 1 diabetes, sex and BMI GRS). Ages shown are the median age (months [mo]) at each visit

The COVID-19 pandemic was associated with an increase in time-varying age-adjusted BMI (*β* = 0.39; 95% CI 0.30, 0.47; *p*<0.001) and with overweight risk at 9 months (*β* = 0.44; 95% CI 0.03, 0.84; *p*=0.034) adjusted for BMI GRS, sex and country of residence (Table 1). Similar associations were observed when BMI SDS or weight-for-length *z* scores were used as the outcome measure (ESM Table 4). No significant interactions between the COVID-19 pandemic and BMI GRS (*p*=0.18), the COVID-19 pandemic and sex (*p*=0.12) or the COVID-19 pandemic and country of residence (*p*=0.44) with respect to overweight risk at 9 months were observed. Moreover, in children for whom all BMI measurements up to 18 months of age were obtained during the COVID-19 pandemic, BMI was higher across all measurements than for children for whom all BMI measurements until the age of 18 months were obtained before the COVID-19 pandemic (*β* = 0.51; 95% CI 0.21, 0.81; *p*=0.001) (Fig. 2).

**Table 1:** Multivariate analysis of the effect of the COVID-19 pandemic and the effect of COVID-19 containment measures during the COVID-19 pandemic (stringency index) on time-varying BMI, BMI at 9 months of age, and risk for overweight (BMI SDS>1) at 9 months of age

	Time-varying BMI (kg/m^2^)				BMI at 9 months of age (kg/m^2^)				Overweight risk (BMI SDS>1) at 9 months of age			
	*N*	*β*	95% CI	Adjusted *p* value	*N*	*β*	95% CI	Adjusted *p* value	*N*	*β*	95% CI	Adjusted *p* value
COVID-19 pandemic	750	0.39	0.30, 0.47	<0.001^a^	745	0.23	−0.02, 0.46	0.052^b^	745	0.44	0.03, 0.84	0.034^b^
Stringency index (per 10-point increase)	738	0.02	0.00, 0.04	0.031^c^	436	0.13	0.01, 0.25	0.033^d^	436	0.23	0.03, 0.43	0.025^d^

For the COVID-19 pandemic analysis, data were analysed across the whole study period; for the stringency index, data obtained during the COVID-19 pandemic were analysed

^a^Adjusted for BMI GRS, sex, age and country of residence

^b^Adjusted for BMI GRS, sex and country of residence

^c^Adjusted for BMI GRS, sex and age

^d^Adjusted for BMI GRS and sex

When restricting the analysis to children followed during the COVID-19 pandemic, the stringency index, a continuous measurement of COVID-19 containment, was positively associated with time-varying BMI (*β* = 0.02; 95% CI 0.00, 0.04 per 10-point increase, *p*=0.031), with BMI at 9 months (*β* = 0.13; 95% CI 0.01, 0.25 per 10-point increase, *p*=0.033) and overweight risk at 9 months (*β* = 0.23; 95% CI 0.03, 0.43 per 10-point increase, *p*=0.025) (Table 1). A stringency index of 88 (the maximum stringency index during the pandemic in this cohort) was associated with a BMI increase of 0.85 kg/m^2^ at 9 months of age compared with a stringency index of 23 (the minimum stringency index during the pandemic in this cohort). Similar associations were observed when using BMI SDS and weight-for-length *Z*-score as outcome measures (ESM Table 4).

### Early-childhood BMI and development of islet autoimmunity

Among the 1050 children, 81 developed islet autoimmunity by 5.5 years of age, including 67 children who developed islet autoimmunity by 2.5 years of age, and 14 children who developed islet autoimmunity between 2.5 and 5.5 years of age. Age-corrected BMI during early childhood was moderately elevated in children who developed islet autoimmunity (HR 1.16; 95% CI 1.01, 1.32; *p*=0.031) (Table 2). Furthermore, overweight risk at 9 months of age was associated with the risk of developing islet autoimmunity up to 5.5 years of age (HR 1.68; 95% CI 1.00, 2.82; *p*=0.048). No significant interactions between overweight risk at 9 months of age and sex or country of residence with respect to islet autoimmunity were observed (*p*=0.23 for overweight risk at 9 months × sex and *p*=0.28 for overweight risk at 9 months × country of residence).

**Table 2:** Cox proportional hazards model for the effect of BMI (time-varying) and overweight risk at 9 months of age on islet autoimmunity up to 5.5 years and up to 2.5 years of age

	Risk for islet autoimmunity during follow-up (up to 5.5 years of age)				Risk for early islet autoimmunity (up to 2.5 years of age)			
	*N*	HR	95% CI	Adjusted *p* value^a^	*N*	HR	95% CI	Adjusted *p* value*
Time-varying BMI (kg/m^2^)	1023	1.16	1.01, 1.32	0.031	1023	1.14	0.98, 1.31	0.088
Overweight risk (BMI SDS>1) at 9 months	1021	1.68	1.00, 2.82	0.048	1021	1.73	0.99, 3.02	0.056

^a^Adjusted for first-degree family history of type 1 diabetes, country of residence, sex and whether BMI was assessed during the COVID-19 pandemic

## Discussion

Using data from the multinational POInT cohort, we found that the COVID-19 pandemic and the stringency of containment measures were associated with higher age-corrected BMI during early childhood and overweight risk at 9 months of age. Early-childhood BMI and overweight risk at 9 months of age were associated with an increased risk of developing islet autoimmunity during childhood.

There is strong evidence that lifestyle factors contribute to the development of overweight, obesity and sub-optimal growth during childhood [19], which is supported by the weight gain observed in school-aged children and adolescents during the COVID-19 pandemic [5, 6]. Our study demonstrated an association between the COVID-19 pandemic and increased BMI, respectively age- and sex-adjusted BMI-SDS, as early as infancy. In addition, we observed that a higher stringency index during the pandemic, reflecting more stringent restrictions imposed by pandemic containment policies, was associated with higher BMI during infancy and early childhood. These results suggest that changes in early environmental exposures in response to containment policies had an impact on children’s BMI. While pandemic-related lifestyle changes toward less favourable dietary and physical activity behaviours were observed primarily in older children [20], it is assumed that such changes already occur in infancy [4, 21]. These include lower rates of exclusive breastfeeding [21], increased consumption of unhealthy snacks, more time spent in restrictive devices and less access to toys [4]. An influence of early feeding practices, such as short breastfeeding duration and an energy-dense/nutrient-poor diet, and low levels of physical activity behaviours on sub-optimal early growth has been demonstrated and reviewed [22–24]. In addition to postnatal lifestyle, COVID-19 pandemic-related alterations of prenatal factors may also have influenced early childhood growth. Indeed, an increase in maternal weight gain during pregnancy has been reported since the onset of the COVID-19 pandemic [25], although studies examining its effect on birthweight have yielded conflicting results [25, 26]. Increased maternal weight gain and birthweight have been shown to be associated with accelerated early growth in previous studies [27–29]. Moreover, in utero exposure to COVID-19 has recently been shown to be associated with accelerated weight gain during infancy [30].

In addition to environmental exposures, genetic factors have previously been found to contribute to early childhood growth, with genetic variants associated with BMI in early childhood differing from those influencing BMI in late childhood and adulthood [17, 31]. We calculated a BMI GRS based on 33 SNPs that have previously been shown to be associated with BMI around the BMI peak and adiposity rebound, the age range that was addressed in the present study. Of note, the observed associations between COVID-19/stringency index and early-childhood BMI and overweight risk in our study were not confounded by the BMI GRS.

Our findings add to the growing body of evidence that links growth patterns during the first year of life to islet autoimmunity risk [9, 10, 32, 33]. A causal role for higher childhood body size on the risk of developing type 1 diabetes has been suggested by two previously published Mendelian randomisation studies [34, 35]. In these studies, childhood body size was assessed at the age of 10 years. Our results, and those from other prospective studies in children at increased risk for type 1 diabetes [9, 10], indicate that body size at a significantly earlier age affects the development of islet autoimmunity. Children who had a BMI SDS>1 at 9 months of age had a significantly increased risk of developing islet autoimmunity during the first 5.5 years of life. The mechanisms related to this association are unknown, but may include beta cell stress as proposed by the accelerator hypothesis [36] and altered immune-cell function and activation through inflammatory or metabolic overload [37, 38].

Our study has several strengths. It was performed across five countries in Europe and with frequent prospective measurement of weight and height in more than 1000 children from as early as 4 months of age, with a nearly equal distribution of children who had their first visits before or during the COVID-19 pandemic. By applying the stringency index of the ‘Oxford COVID-19 Government Response Tracker’ (OxCGRT), which assessed pandemic containment measures on a daily basis in all participating countries, we were able to assess the impact of COVID-19 containment measures, despite differences in policy responses between participating countries. Our study is limited by the lack of prenatal data, such as gestational weight gain and in utero exposure to COVID-19, birthweight information and measurements before 4 months of age. Therefore, modelling of BMI curves to determine the age and BMI at peak was not possible. Instead, we used BMI measurements at 9 months of age as a growth parameter; this measurement has been previously proposed as a surrogate marker for peak BMI [18]. Furthermore, we were unable to examine whether the observed increase in BMI during the pandemic was related to changes in lifestyle behaviour, as such data were not collected in our study. Because the POInT trial is still unblinded, we were unable to examine whether the oral insulin intervention affected the observed associations. However, in the Pre-POInT early trial, which included children at the same age, oral insulin administration did not affect blood glucose, insulin and C-peptide values compared with placebo [39]. Moreover, as the treatment is randomised and the children were receiving treatment before and during the pandemic, we do not expect that this intervention would affect the findings. Our results suggest that the observed associations are not dependent on sex, but may not be representative of children from other ethnic and racial groups, as they were generated in European children.

In conclusion, our study showed an increase in early-childhood BMI in children followed during the COVID-19 pandemic, which was associated with the stringency index during the pandemic period. Further studies are warranted to clarify whether lifestyle changes during pregnancy and infancy in response to containment policies led to the observed increase in BMI. In agreement with previous reports, we observed that accelerated early growth was associated with the development of islet autoimmunity in children with genetic susceptibility to type 1 diabetes.

### Supplementary Information

Below is the link to the electronic supplementary material.

Supplementary file1 (PDF 394 KB)

## Data Availability

Data will be available on submission of a signed transfer agreement; please email cc@gppad.org and the corresponding author.

## Abbreviations

GPPAD: Global Platform for the Prevention of Autoimmune Diabetes
GRS: Genetic risk score
POInT: Primary Oral Insulin Trial
SDS: Standard deviation score
