# Supplementary material for: Early-childhood body mass index and its association with the COVID-19 pandemic, containment measures and islet autoimmunity in children with increased risk for type 1 diabetes
Source: Diabetologia. 2024 Jan 12;67(4):670–8. doi: 10.1007/s00125-023-06079-z (PMC10904508; doi:10.1007/s00125-023-06079-z)
Supplement: Supplementary file 1 — Supplementary file1 (PDF 394 KB) [file 125_2023_6079_MOESM1_ESM.pdf]

Supplemental Material

Early childhood body mass index and its association with COVID-19 pandemic, containment measures, and islet autoimmunity in children with increased risk for type 1 diabetes

S. Hummel et al.

ESM Methods 1	Genotyping
ESM Methods 2	Definition of islet autoimmunity outcome
ESM Table 1	Number of children followed before and during COVID-19 pandemic, and their median age (IQR) per visit.
ESM Table 2	SNPs used to calculate the BMI genetic risk score according to their association with early childhood BMI
ESM Table 3	Demographics and characteristics of the study population
ESM Table 4	Multivariate analysis on the effect of COVID-19 pandemic and on the effect of COVID-19 containment measures (Stringency Index) during COVID-19 pandemic on time-varying BMI-SDS, weight-for-length Z-Score and overweight (weight-for-length Z-score >2) at 9 months of age
ESM Figure 1	BMI in relation to age. Spaghetti plot on 8839 longitudinal BMI measurements of 1050 children.
ESM Appendix	Acknowledgements

ESM Methods 1. Genotyping

SNP data were generated as previously described (14) using the Infinium Global Screening Array (version 3.0, Illumina Inc.) performed on DNA extracted from dried blood spots of children for whom consent to store and use dried blood spots for additional research was provided. Samples were excluded if the genotype call rate was less than 95%, if there was a mismatch between genotyped sex and reported sex, or if there was an outlying heterozygosity rate (>3 SD at a minor-allele frequency [MAF] of <1% or MAF of ≥1%). Variants were filtered if the call rate was less than 98% or if the MAF was greater than 1%. Imputation of additional variants was performed using the Sanger Imputation Service (https://imputation.sanger.ac.uk/) and the Haplotype Reference Consortium reference panel (http://www.haplotype-reference-consortium.org/) HRC r1.1 2016 (GRCh3/hg19).

ESM Methods 2. Definition of islet autoimmunity outcome

Islet autoantibodies were measured centrally at 2 independent GPPAD Core laboratories, located at the Institute of Diabetes Research, Helmholtz Munich, Germany, and at the University of Bristol Medical School, Diabetes and Metabolism, Learning and Research, Southmead Hospital, Bristol, United Kingdom (for confirmation of results). Serum samples from each visit were measured for autoantibodies against insulin (IAA), glutamate decarboxylase-65 (GADA), insulinoma-associated antigen-2 (IA-2A), and zinc transporter-8 (ZnT8A) at the German laboratory. IAA were detected using a competitive radiobinding assay (RBA) with protein A/G immunoprecipitation and 125I-labeled recombinant human insulin. GADA and IA-2A were measured based on the National Institute of Diabetes and Digestive and Kidney Diseases (NIDDK) harmonized assay protocol using 35S-methionine–labeled antigens produced by in vitro transcription and translation of N-terminally truncated GAD65 (amino acids 96–585) or IA-2ic (amino acids 606–979), encoded in the pTNT plasmid vector (Promega), as previously described. For GADA, ELISA (RSR Ltd.) was used as the second test if the RBA result was positive. ZnT8A was tested in separate assays to detect autoantibodies to the arginine 325R and tryptophan 325W human ZnT8 variants (ZnT8RA and ZnT8WA, respectively). Assays were performed using 35S-methionine–labeled in vitro

transcribed/translated recombinant ZnT8 (amino acids 268–369), as previously described. Children were classified as ZnT8A positive if they were positive for ZnT8RA and/or ZnT8WA or as ZnT8A negative if the tests were negative for both antibody specificities. These RBAs had sensitivities and specificities of 54% and 99% for IAA, 66% and 99% for GADA, 76% and 100% for IA-2A, 56% and 99% for ZnT8RA, and 50% and 99% for ZnT8WA according to the Islet Autoantibody Standardization Program (IASP) 2016 Workshop. Samples that tested positive for islet autoantibodies at the Munich laboratory were sent to the second central autoantibody laboratory in Bristol for confirmatory testing. Here, IAA were assayed using a competition RBA with 125I-labeled human insulin, as previously described. GADA and IA-2A were assayed using the NIDDK harmonized assay protocol using 35S-methionine–labeled in vitro transcribed/translated recombinant full-length GAD65 or IA-2ic. ZnT8RA and ZnT8WA were tested in separate RBAs based on the NIDDK harmonized assay protocol. These RBAs had sensitivities and specificities of 54% and 99% for IAA, 74% and 97% for GADA, 70% and 100% for IA-2A, 60% and 100% for ZnT8RA, and 46% and 100% for ZnT8WA according to the IASP 2015 Workshop. If a sample tested positive for a specific autoantibody by tests at both laboratories, a subsequent sample was tested by both laboratories to confirm persistence of the islet autoantibody–positive status for the detected autoantibody.

ESM Table 1. Number of children followed before and during COVID-19 pandemic, and their median age (IQR) per visit.

**Visit number**	**Children before/ during COVID-19 (*n*)**	**Age (years) Median (IQR)**
**Visit 1**	612/436	0.51 (0.45; 0.54)
**Visit 2**	487/513	0.67 (0.61;0.71)
**Visit 3**	412/558	0.83 (0.78;0.87)
**Visit 4**	293/686	1.16 (1.10;1.20)
**Visit 5**	189/760	1.50 (1.49;1.51)
**Visit 6**	64/876	2.00 (1.97;2.02)
**Visit 7**	12/926	2.49 (2.47;2.51)
**Visit 8**	0/768	3.00 (2.98;3.02)
**Visit 9**	0/565	3.50 (3.45;3.53)
**Visit 10**	0/355	3.99 (3.95;4.03)
**Visit 11**	0/209	4.50 (4.44;4.54)
**Visit 12**	0/94	5.00 (4.95;5.03)
**Visit 13**	0/23	5.47 (5.44;5.51)

ESM Table 2. SNPs used to calculate the BMI genetic risk score according to their association with early childhood BMI [(1)]

Name	SNP	Risk allele	Allele frequency in POInT cohort	Allele weight	Cluster acc. to (1)
LEPR	rs10493377	A	53%	0.057	Transient
LEPR	rs10889551	G	66%	0.088	Transient
LEPR	rs2767486	G	18%	0.143	Transient
TNNI3K	rs10493544	T	45%	0.054	Early rise
NR5A2	rs2816985	G	49%	0.059	Transient
AC105393.2	rs77165542	C	98%	0.187	Early rise
ADCY3	rs11676272	G	44%	0.089	Early rise
LCORL	rs2610989	T	26%	0.060	Early rise
HHIP	rs1032296	T	35%	0.052	Transient
PCSK1	rs6899303	C	63%	0.057	Transient
PCSK1/CAST	rs263377	A	43%	0.054	Transient
GLP1R	rs2268657	T	58%	0.056	Transient
GLP1R	rs2268647	T	50%	0.048	Transient
GLP1R	rs1820721	A	51%	0.061	Transient
UBE3D	rs209421	G	26%	0.073	Transient
OPRM1	rs1772945	A	51%	0.056	Transient
MLXIPL	rs17145750	C	84%	0.070	Transient
LEP	rs10487505	C	47%	0.056	Early rise
KLF14	rs287621	T	30%	0.064	Transient
KLF14	rs12672489	C	77%	0.067	Early rise
HNF4G	rs117212676	A	3%	0.166	Early rise
PTCH1	rs28457693	G	12%	0.073	Transient
PLCE1	rs1830890	G	33%	0.067	Early rise
SCGB1A1	rs1985927	C	71%	0.060	Early rise
EHBP1L1	rs2298615	T	22%	0.071	Transient
RP11-405A12.2	rs2728641	C	47%	0.050	Transient
RP11-690J15.1	rs6538845	C	47%	0.055	Early rise
NCOR2	rs3741508	T	86%	0.083	Transient
SH3GL3	rs2585058	G	48%	0.063	Transient
KIAA0895L	rs111810144	T	3%	0.147	Early rise
RIN2	rs148252705	T	96%	0.157	Transient
EFCAB8	rs13038017	C	55%	0.054	Early rise
PTCHD1-AS	rs5926278	T	1%	0.149	Transient

The risk score was calculated by multiplying the number of risk alleles (i. e. 0, 1 or 2 for each single SNP) with the weight assigned to each SNP and then summing up the weighted contributions of all SNPs. As an example, the risk score for a child, homozygous for the risk allele of rs13038017 (weight 0.054), heterozygous for the risk allele of rs148252705 (weight 0.157), homozygous for the non-risk allele of rs1830890 (weight 0. 067) and for all other SNPs in the BMI GRS is calculated as follows: BMI GRS = (2 * 0.054) + (1 * 0.76) + (0 * 0. 0670.76) + 0 = 0.868

1) Helgeland Ø, Vaudel M, Sole-Navais P, Flatley C, Juodakis J, Bacelis J et al. Characterization of the genetic architecture of infant and early childhood body mass index. Nature metabolism 2022;4:344–58

ESM Table 3. Demographics and characteristics of the study population

Variable	*n* (%)	Median (IQR)
**Children included**	1050	
**Male**	533 (50.8%)	
**Female**	517 (49.2%)	
**Age at enrolment (years)**		0.51 (0.45; 0.54)
**Follow-up after enrolment (years)**		2.9 (2.4; 3.5)
**FDR**		
**No**	494 (47.0%)	
**Yes**	556 (53.0%)	
**Country**		
**Germany**	504 (48.0%)	
**Munich**	276 (26.3%)	
**Dresden**	141 (13.4%)	
**Hanover**	87 (8.3%)	
**Poland**	242 (23.0%)	
**Sweden**	173 (16.5%)	
**Belgium**	80 (7.6%)	
**United Kingdom**	51 (4.9%)	
**BMI risk - SNP data available**	752 (71.6%)	
**BMI GRS, continuous**		2.40 (2.25; 2.57)

FDR, first-degree family history with type 1 diabetes; BMI GRS, BMI genetic risk score; SNP, single-nucleotide polymorphisms

ESM Table 4. Multivariate analysis on the effect of COVID-19 pandemic and on the effect of COVID-19 containment measures (Stringency Index) during COVID-19 pandemic on time-varying BMI-SDS, weight-for-length Z-Score and overweight (weight-for-length Z-score >2) at 9 months of age.

	**Time-varying BMI-SDS**				**Weight-for-length Z-score at 9 months of age**				**Overweight (weight-for-length Z-Score>2) at 9 months of age**			
	N	ß	95% CI	Adjusted *p*-value	N	ß	95% CI	Adjusted *p*-value	N	ß	95% CI	Adjusted *p*-Value
**Whole study period**												
COVID-19 pandemic	750	0.37	(0.31; 0.43)	<0.001*	745	0.15	(-0.003; 0.30)	0.055*	745	0.90	(0.08; 1.72)	0.030*
**During COVID-19 pandemic**												
Stringency Index (per 10 units increase)	738	0.02	(0.01; 0.03)	0.002†	436	0.09	(0.01;0.17)	0.026†	436	0.37	(0.01; 0.73)	0.048†

*adjusted for BMI GRS and country of residence, †adjusted for BMI GRS

ESM Figure 1. BMI in relation to age. Spaghetti plot on 8839 longitudinal BMI measurement of 1050 children.

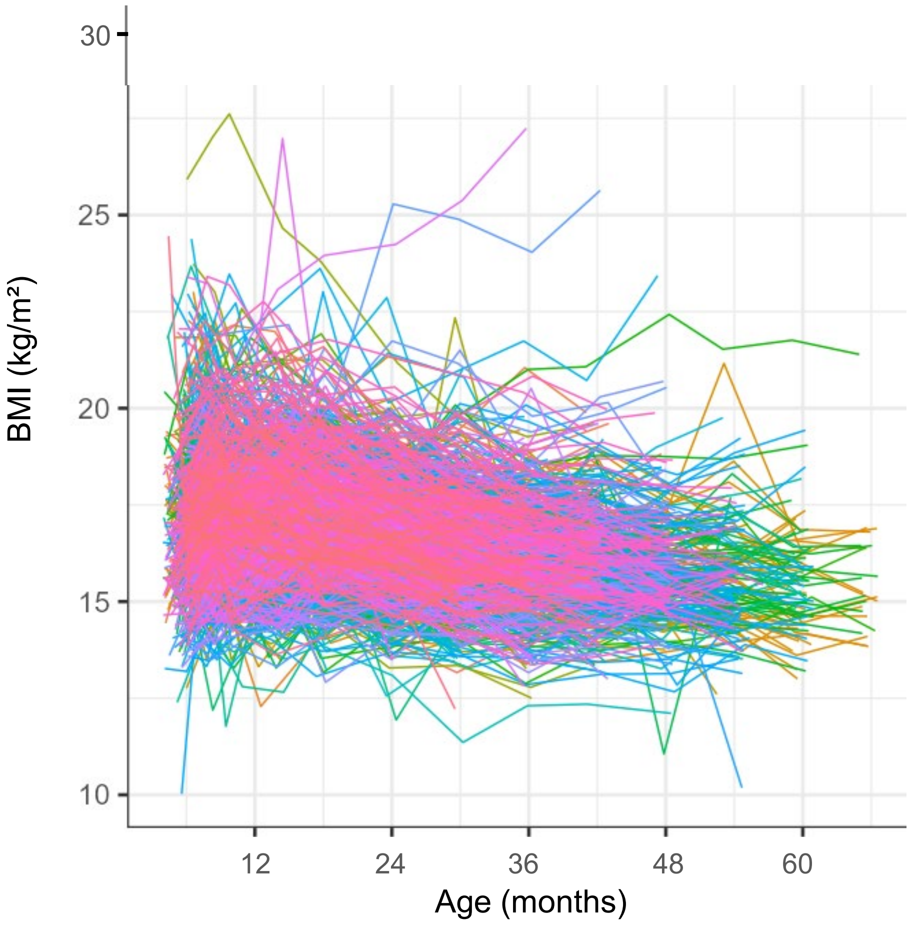

ESM Appendix: Acknowledgements

GPPAD Coordinating Centre: Melanie Gündert, Stefanie Arnolds, Karina Blasius, Michaela Dilong, Nadine Friedl, Gertrud Göppel, Florian Haupt, Martin Heigermoser, Maja Hergl, Bianca Höfelschweiger, Krisztian Kisfügedi, Nadine Klein, Stefanie Löbner, Rebecca Niewöhner, Marlon Scholz, Lena Schwenker, Katharina Schütte-Borkovec, Andreas Weiß, José Maria Zapardiel Gonzalo, Lorena Wendel

Belgium Clinical Centre: Kristina Casteels, Jasmin Paulus, Brontë Vrancken, Charlien Jannsen, Anne Rochtus, An Jacobs, Hilde Morobé, Natalie Van den Driessche, Renka Van Heyste, Janne Houben, , Veerle Vanhuyse.

Germany, Dresden Clinical Centre: Ezio Bonifacio, Reinhard Berner, Sari Arabi, Lisa Barbknecht, Sevina Dietz, Franziska Ehrlich, Gita Gemulla, Zahra Gholizadeh, Raphael Hoffmann, Angela Hommel, Franziska Lange, Anja Loff, Robert Morgenstern, Anne Schille, Maike Sigg, Marc Weigelt, Andre Weise, Nicole Zubizarreta.

Germany, Hanover Clinical Centre: Olga Kordonouri, Thomas Danne, Laura Galuschka, Carolin Kruse, Sarah Landsberg, Karin Lange, Erika Marquardt, Felix Reschke, Frank Roloff, Thekla von dem Berge, Jantje Weiskorn, Mareike Niemeyer, Bianca Schmidt

Germany, Munich Clinical Centre: Anette-G. Ziegler, Peter Achenbach, Evangelia Asvou, Melanie Bunk, Nicole Bücheler, Anna Hofelich, Elisabeth Huber, Stefanie Jacobson, Janna Jaud, Melina Kaiser, Alexandra Käßl, Benjamin Marcus, Annette Munzinger, Franziska Reinmüller, Katharina Sarcletti, Sarah Schmidt, Franka Teichgräber, Veronika Vollmuth, Merve Vurucu, Christiane Winkler

Poland Clinical Centre: Agnieszka Szypowska, Mariusz Ołtarzewski, Sylwia Dybkowska, Lidia Groele, Dorota Owczarek, Katarzyna Popko, Adrianna Cieloch, Elżbieta Górska, Agnieszka Mroczek, Beata Zduńczyk, Anna Zych, Wiktoria Czerwińska, Natalia Dziedzic

Sweden Clinical Centre: Helena Elding Larsson, Markus Lundgren, Daniel Agardh, Sofie Alström Mortin, Rasmus Bennet, Charlotte Brundin, Susanne Dahlberg, Lina Fransson, Malin Goldman-Tsubarah, Maria

Hyllengren, Ida Jönsson, Marielle Lindström, Jessica Melin, Hannah Nenonen, Anita Ramelius, Kobra Rhamati, Falastin Salami, Hanna Samuelsson, Carina Törn, Ulrika Ulvenhag

United Kingdom, Newcastle Clinical Centre: Catherine Owen, Steve Robson, Helen Gallon, Laura Gebbie, Fiona Jenkinson, Sharon King, Steven Pratt, Claire Simmister, Evelyn Thomson, Eileen Walton, Andrew Johnston, Fiona Yelnoorkar, Stephen Crulley, Caroline Miller

United Kingdom, Oxford Clinical Centre: Rachel Besser, John A Todd, Matthew Snape, Sophia Hawkins, Yama Farooq, Ian Smith, Helen Ratcliffe, Fenella Roseman, Hannah Robinson, Nazia Taj, Sophie Vernon, Conor Whelan, Tabitha Wishlade

Data Safety and Monitoring Board: Polly Bingley (University of Bristol, Bristol, UK), Ulrich Heininger (Division of Paediatric Infectious Diseases and Vaccinology, University Children's Hospital, Basel, Switzerland), Markus Pfirrmann (Institut für Medizinische Informationsverarbeitung, Biometrie und Epidemiologie (IBE), Ludwig-Maximilians-Universität, Munich, Germany), Wolfgang Rascher (Department of Pediatrics and Adolescent Medicine, Erlangen, Germany), Paul Turner (Nuffield Department of Medicine, Medical Science Division, Oxford, UK)

Beta-cell Autoantibody and Confirmatory Laboratory: Bristol Medical School, The University of Bristol, Bristol, UK

Genotyping Laboratory: Grace London, LGC Ltd., Hertfordshire, UK

Electronic Questionnaires/ Database/ Pharmacovigilance: PHARMALOG, GmbH, Ismaning, Germany

IMP/ Study Product Supply: Donation from Eli Lilly Product Research and Development (PR&D), Eli Lilly Indianapolis Active Pharmaceutical Ingredient (IAPI), Indianapolis, USA

Allphamed Pharbil Arzneimittel GmbH, Göttingen, Germany

Infinant Health Davis, California, USA

Pharmacy of the University Hospital of Heidelberg, Germany

Biorepository: Integrated BioBank of Luxembourg (IBBL), Dudelange, Luxembourg

IVRS: GxP brain GmbH, Berlin, Germany
